# Examining the Impact of School Esports Program Participation on Student Health and Psychological Development

**DOI:** 10.3389/fpsyg.2021.807341

**Published:** 2022-01-24

**Authors:** Michael G. Trotter, Tristan J. Coulter, Paul A. Davis, Dylan R. Poulus, Remco Polman

**Affiliations:** ^1^Faculty of Health, School of Exercise and Nutrition Sciences, Queensland University of Technology, Brisbane, QLD, Australia; ^2^Department of Psychology, Umeå University, Umeå, Sweden; ^3^School of Health and Human Sciences, Southern Cross University, Bilinga, QLD, Australia

**Keywords:** competitive video games, self-regulation, positive youth development, health, sport, esports, growth mindset

## Abstract

This study examined the influence of 7 high school esports developmental programs on student self-regulation, growth mindset, positive youth development (PYD), perceived general health and physical activity (PA), and sport behaviour. A total of 188 students (male *n* = 120; female *n* = 68) originally participated (89 enrolled in an esports program in their school and 99 acted as aged-matched controls), with 58 participants (*n* = 19 esports group; *n* = 39 controls) completing both pre- and post-program information. At baseline, no significant differences were found between youth e-athletes and their aged-matched controls. The analysis for the observation period showed a significant interaction effect for the PYD confidence scale, with *post-hoc* comparisons showing a significant decrease in the control group from pre- to post assessment whereas the esports group remained the same. Time main effects showed a decrease in the self-regulation motivation factor, PYD connection factor and PA for all participants. Overall, this study showed that students enrolled in their respective school esports program did not differ from those who did not in self-regulation, growth mindset, PYD, perceived health and PA, and sport behaviour. It was likely that all participants showed a decrease in motivation, connection, and PA due to COVID19 lockdown during the study period. This study is the first to investigate the longitudinal impact of student involvement in high school esports and showed that esports participation did not have a negative impact on any health or psychological factors.

## Introduction

Esports has become hugely popular with the number of players and spectators growing year on year (Newzoo, 2020). The esports industry has been valued at over 24 billion dollars (Ahn et al., 2020) and professional esports athletes (e-athletes) can earn as much (or more) than traditional athletes (Finance Monthly, 2018). The rapid growth in esports is mostly due to the high levels of engagement of young people. A recent industry report showed that almost half of all esports fans are aged between 13 and 24 years (Nielsen, 2019). There has also been a 500% increase in the number of high school esports developmental and grassroots programs and tournaments in the USA between 2018 and 2019 (Hennick, 2019). The increased popularity in esports has been accompanied by several concerns regarding sedentary behaviour, psychological development, and physical and psychological wellbeing (e.g., Shum et al., 2021).

Many esports leagues and organisations operate similarly to other sports; universities have begun to include esports as part of their sports portfolios and, philosophically, esports meets nearly every criteria for traditional sport (Holden et al., 2017; QUT, 2021). Furthermore, researchers are increasingly accepting esports as a sport (Jang and Byon, 2019). In traditional sport, the activity itself, as well as the social structure of sport, has been shown to result in improved mental and physical well-being (e.g., Eime et al., 2015). These factors have also provided opportunities for self-regulatory skill development (e.g., Collins and Durand-Bush, 2014) and positive youth development (Holt, 2016). Early evidence in esports research suggests similar trends. For example, in a qualitative study of CS:GO players Nielsen and Hanghøj (2019) provided preliminary evidence that grassroots esports programs can result in psychological skills development, which have the potential to transfer to everyday life. Conversely, in a cross-sectional study of e-athletes Trotter et al. (2021) reported evidence that the organisational structure of esports provide less opportunities for the development of social support, self-regulatory skills, psychological skills, and physical activity (PA) behaviour. Overall, there is a need to examine the potential of community or school esports programs on psychological development, health, and PA behaviour, especially in adolescent populations.

Despite negative connotations about video gaming (Mihara and Higuchi, 2017) and esports (Shum et al., 2021), a review by Granic et al. (2014) suggested that playing video games was associated with cognitive, motivational, social, and health benefits. Similarly, it has been theorised that pedagogical supervision in adolescent esports programs can assist in identifying potential signs of problematic gaming (Wimmer et al., 2021). Such esports programs are becoming increasingly common in school curriculum in the USA (Hennick, 2019). Currently, there is limited empirical evidence on the associated benefits of participation in adolescent esports programs. However, research indicates that such programs have the potential to positively impact the development of communication, teamwork, and problem-solving skills (Rothwell and Shaffer, 2019), professional and academic skills, social and emotional learning (Reitman et al., 2020), social belonging and mental health (Tjønndal and Skauge, 2020). In addition, only a small proportion of students enrolled in these programs have report problematic gaming behaviours (e.g., gaming addiction; Ortiz de Gortari, 2019). Despite this evidence, no study examining youth esports have used a longitudinal research design. As such, it is not possible to draw conclusions about the causal relationship participation in such program has on student development.

### Self-Regulation

Previous research has suggested that metacognitive functions may theoretically be important for preventing the development of problematic gaming behaviours from involvement in esports programs (Brevers et al., 2020). In a review, Brevers et al. (2020) suggested there is a need to identify markers (e.g., self-regulation) that delineate high involvement from problematic esports engagement, as well as a better understanding of how such self-regulatory processes unfold among esports athletes.

To date, no literature exists on the self-regulatory processes in adolescent e-athletes. Previous research with adult esports athletes suggests that developmental esports programs may offer an avenue for the development of self-regulation (Trotter et al., 2021). Moreover, studies on the development of self-regulation in traditional adolescent athletes report that modelling by parents and coaches is predictive of an adolescent athlete belonging to an elite group (Teques et al., 2019). Similarly, in a longitudinal study of elite youth soccer players Erikstad et al. (2018) showed that high self-regulated adolescent traditional athletes were more likely to be selected for national initiatives and that sports participation may contribute to differences in self-regulatory skills. Despite research indicating the relationship between sporting and academic performance and self-regulation (Jonker et al., 2010a), to date, it is unclear if self-regulation predicts performance in esports or if adolescent esports programs impact the development of self-regulation. This study seeks to extend previous work by examining the use of self-regulatory skills in adolescent e-athletes enrolled in a high school esports program.

### Positive Youth Development

Previous research has suggested that sport is a globally recognised domain in which positive youth development (PYD) can be successfully promoted (Holt, 2016). PYD is conceptualised as a strength-based method of developing adolescents and children's personal resources, rather than seeing adolescents and children as problems to be solved (Bruner et al., 2021). A recent meta-analysis of 29 sport based PYD interventions found that these interventions can be effective at improving PYD outcomes (Bruner et al., 2021). With esports programs in schools on the rise, it is important to determine if similar youth esports programs will have the same effect on youth development as sporting programs. Understanding the impact of such esports programs is important, as previous research suggests that co-curricular video gaming could be linked with high time costs, physical injuries and problematic psychological functioning (Shum et al., 2021). Similarly, other critics of adolescent video game usage have raised concerns about poor nutrition, decreased PA levels, and potential behavioural disorders related to increased video game usage (Balatoni et al., 2020). Esports programs, on the other hand, might offer an opportunity to engage adolescents who may not be so interested in traditional sports clubs (Tjønndal and Skauge, 2020) and, thus, miss out on the important developmental opportunities available in traditional sports, such as a growth mindset (Lauer et al., 2018).

### Growth Mindset

Previous research suggests that developing a growth mindset is critical to elite adolescent development strategies in sport (Lauer et al., 2018) and academia (Burnette et al., 2013). A growth mindset has been described as the belief that talent can be developed through hard work, good strategies, and input from others (Dweck, 2016), and is not dissimilar to a task or mastery orientation (Gilbert et al., 2010)—both of which are associated with intrinsic motivation and effort (Kim and Gill, 1997). Himmelstein et al. (2017) suggested that a growth mindset is a strategy used by adult esports athletes for successful performance. Currently, no research has explored a growth mindset in adolescent esports athletes. This study will determine levels of a growth mindset among adolescent esports athletes enrolled in a high school esports program.

### Physical Activity

Despite adult e-athletes reporting that PA behaviour was the least important factor contributing to their performance (Railsback and Caporusso, 2019), research has shown that increased PA behaviour is associated with higher in game ranking (Trotter et al., 2020). The research on PA behaviour in e-athletes is equivocal. The percentage of e-athletes who exercise to specifically increase their in-game performance is between 6 and 9% (Pereira et al., 2019). Adult e-athletes, instead, appear to be motivated to exercise to maintain their general health (Kari and Karhulahti, 2016). However, some League of Legends players have been reported to exercise 4.2 times per week, more than most Americans who exercise 3 times per week (Thomas et al., 2019). The perception among adult e-athletes that PA is not important for performance may be due to the previous lack of developmental esports programs. Current research suggests that PA is becoming a normal part of high school esports program, with high schools in Austria, Norway and the USA including regular PA as part of their esports programs (Rothwell and Shaffer, 2019; Tjønndal and Skauge, 2020; Wimmer et al., 2021). This study seeks to build on the findings of previous literature by exploring the frequency of which e-athletes enrolled in a high school esports program are physically active and compare their PA levels with an aged-matched control group.

### Perceived General Health

There is currently little empirical research on the association between esports and health. One study in adult e-athletes showed that in game rank was not associated with ratings of self-perceived health (Trotter et al., 2020). There is some evidence from the gaming literature which showed that heavy video gaming in young adults was associated with not meeting World Health Organisation PA guidelines (World Health Organization, 2018) and lower scores on indicators of general health compared to the general population (King and Delfabbro, 2009). However, to date, no research has explored the relationship between perceived general health and adolescent e-athletes engaged in a school esports development program.

### Study Aims

The current study was exploratory in nature and had two aims:

Study aim 1, to explore differences between student e-athletes enrolled in a school based esports programme and an aged-matched control group on self-regulation, growth mindset, PYD, PA behaviour, and self-perceived health.
Study aim 2, to explore the effect of the school esports programs on self-regulation, growth mindset, PYD, PA behaviour and self-perceived health.

Considering most esports psychology literature has focused on the performance or health of adult e-athletes (Poulus et al., 2020, 2021a,b; Trotter et al., 2020, 2021). This study therefore will investigate the psychology and health of adolescent e-athletes. The study was conducted with adolescent males and females aged 13–18 years old. There are important differences in maturation between males and females. This is reflected in gender differences in self-regulation (e.g., Silverman, 2003), PA behaviour (Corder et al., 2019), and self-reported health (Potrebny et al., 2017). In addition, esports is more likely to played be males than females (Andrews and Crawford, 2021). Similarly, calendar age in adolescence is associated with differences in self-regulation (Steinberg et al., 2018), PYD (Taylor et al., 2017), PA behaviour (Corder et al., 2019), and indirectly to health status (Granger et al., 2017). Therefore, this study controlled for both age and gender.

## Methods

### Participants

A total of 188 high school students (male *n* = 120; 63.7%); female *n* = 67; 37.3%) aged between 13 and 18 years, from 7 private schools in Queensland, Australia, took part in this study. All students attended a school participating in a local high school esports tournament, with a dedicated esports training program. Of the 188 students, 89 (76 males, 13 females; Average age = 14.28 years) played esports for their school, and the remaining 99 (44 males, 55 females; Average age = 14.57 years) were included in a aged-matched control group. The control group in the present study consisted of participants of similar age but who did not participate in the esports program. In addition, control participants were recruited from all participating Schools.

Of the 188 participants, 58 male (*n* = 22; 37.9%) and female (*n* = 36; 62.1%) students completed both the pre-test and the post-test survey, representing 6 of the 7 schools. A 69.15% dropout rate was observed, primarily due to COVID-19 restrictions in the greater Brisbane area during the study period. Of the 58 remaining students, 19 played esports for their school and 39 were aged-matched controls.

### Measures

#### Self-Regulation

Self-regulation was measured using the Trait Self-Regulation Questionnaire (TSRQ; Hong and O'Neil, 2001). The TSRQ has been used previously in adolescent sport to examine self-regulation (Toering et al., 2009, 2012; Jonker et al., 2010a; e.g., Jonker et al., 2010b) and consists of 32 items and two higher-order factors—metacognition and motivation. Metacognition is represented by two lower-order factors—planning and self-checking. Planning refers to behaviours related to the planning of goals and strategies involved in self-regulation (e.g., “*I determine how to solve a task before I begin”*). Self-checking refers to behaviours related to the monitoring and reflection phases of self-regulation (e.g., “*I cheque my work while I am doing it”*). Motivation is also represented by two lower-order factors—effort and self-efficacy. Effort refers to the amount of effort expended on attaining desired goals (e.g., “*I am willing to do extra work on tasks to improve my knowledge.”*). Self-efficacy refers to self-beliefs in one's ability to complete a task (e.g., “*I am confident I can understand the most complex material presented by the teacher in this course.”*). The higher-order factors of metacognition and motivation load onto the overall factor of trait self-regulation. The TSRQ is scored on a 4-point Likert scale ranging from 1 (*almost never)* to 4 (*almost always*). In a study of Korean high school students Hong and O'Neil, (2001) provided evidence for the hierarchical structure of the TSRQ, whereas (Jonker et al., 2010a) provided evidence for its satisfactory construct validity.

The initial confirmatory factor analysis (CFA) of the 3^rd^ order factorial structure did not provide an adequate fit for the TRSQ. Modification indices indicated to cross correlate the error term of item 26 with the error term of item 28 (both self-efficacy factor) and the error term of item 18 with the error terms of items 19 and 23 (all effort factor). The subsequent CFA provided an adequate fit for the model. (χ^2^ = 742; *P* < 0.01; RMSEA = 0.058; TLI = 0.91; CFI = 0.91; GFI = 0.81 and Pclose = 0.05), with all indices, except GF, meeting minimal fit requirements. Finally, the Cronbach alpha for the scale as a whole at baseline was excellent (α = 0.95), as well as for the individual factors planning (α = 0.87), self-checking (α = 0.86), effort (α = 0.85) and self-efficacy (α = 0.92).

#### Growth Mindset

The Self-Theories Questionnaire (STQ) was used to measure participants' growth mindset (Dweck, 2000). Growth mindset is; an individual's beliefs that their talents can be developed through hard work, coaching, or good strategies (Dweck, 2016). The STQ has 6-items (e.g., “*You have a certain amount of intelligence, and you really can't do much to change it”*) and is scored on a 4-point scale, ranging from 1 (*disagree*) to 4 (*agree*).

The initial CFA did not provide an adequate fit for the STQ. Modification indices indicated to cross correlate the error term of item 5 with the error term of items 1, 4 and 6. The subsequent CFA provided an excellent fit for the model. (χ^2^ = 4.09; *P* = 0.54; RMSEA = 0.001; TLI = 1.00; CFI = 1.00; GFI = 0.99 and Pclose = 0.75), with all indices meeting fit requirements. Cronbach alpha for the scale for the baseline data was good (α = 0.89).

*PYD*: The Very Short Measure of PYD (VSMPYD) was used to measure participants' positive youth development (Geldhof et al., 2014). The VSMPYD measures five factors of PYD (Caring, Competence, Confidence, connection, and Character). Côté and Erickson's (2016) theory of PYD in sport suggests that only four of the five PYD factors are relevant in sport (Competence, Confidence, connection, and Character), and recommended removing the items for the Caring factor from questionnaires. However, as PYD has not been explored in esports, all five factors of PYD were included in this study. The items were measured using several different 5-point Likert scales. The VSMPYD has demonstrated good reliability with video gamers (Hilliard et al., 2018) and high school students (Travers and Mahalik, 2019). Due to the different response sets in the VSMPYD, no CFA or reliability analysis were conducted.

#### PA and Sport Participation

Based on the Australian PA guidelines for children and adolescents aged 5–17 of at least 60 mins of daily PA (World Health Organization, 2018), the following one-item question was used using an 8 point likert scale: “How many days are you physically active for longer than 60 mins?”. Participants were asked to indicate the number of days they were physically active on a scale ranging from 0 (none) to 7 (seven). In addition, the participants were asked about their sport engagement. Specifically, (i) Do you play sports; and (ii) if yes, on average, how many minutes of sport do you participate in per week?

#### General Health

The SF-1 (Avery et al., 2006) was used to measure general health. The SF-1 is the short form of the SF-36, which is a commonly used generic measure of people's general health status (Ware, 2000; Avery et al., 2006). The SF-1 has one item, where participants are asked to rate their health on a scale ranging from 1 (*poor*) to 5 (*excellent*). The SF-1 has been used previously by the South Australian Government (Avery et al., 2006) to measure perceived general health. The SF-1 has been shown to predict health behaviours, including PA and weight status (Segovia et al., 1989), and mortality and morbidity (Benyamini and Idler, 1999).

### Procedure

This study was approved by a University's Research Ethics Committee (approval number 1900000790). A convenience sampling approach was used for data collection, a previous existing relationship between a member of the Anglican Schools Commission and the lead researcher allowed for introductions to participating schools. All participating schools were recruited through the Anglican Schools Commission Office, which coordinated the competition and program. Each participating school ran its own esports program independently, choosing how to organise training sessions and the time, frequency and duration of these sessions. Each school had one teacher who was responsible for coordinating all esports related activities. It was not possible to observe the specific training activities for each school individually. The coordination of competitions between schools was organised at the end of each term and held at the Queensland University of Technology over the course of a day.

Permission from each participating school was obtained from the school Principal and each student provided informed consent prior to undertaking the survey. The teacher responsible for coordinating the esports team at each school distributed the survey to all students and explained the process of consent. All students (including control participants) completed the survey during the time allocated for esports training at the participating schools, under the teacher's supervision. Once consent was granted, all students completed the survey online using school computer resources, which took ~20–30 mins.

The competitive season ran from term 1 through to term 2 (i.e., late January to late June, 2020). Once the competitive season started, participants from the esports group took part in training sessions run at the participating school. These training sessions occurred between 1 and 2 times per week, for 8 weeks, during terms 1 and 2 at each school. Training sessions typically involved the development of game-based strategies, team bonding, and playing practise games. In each school term, there was one live competition held at the Queensland University of Technology (QUT) esports arena facility. Competition days were run similarly to professional esports events, with live streaming, a dedicated competition space, and participating students and teachers as a live audience. Data collection occurred in two, two-week periods. Participants completed the pre-survey questionnaire prior to the beginning of the commencement of training, (between the 10th and 23rd of February) and the post-test survey after all competitions had ceased in term 2 (between the 8th and 22nd of June).

### Analysis Strategy

Data was screened for outliers and any incomplete surveys were removed from the data set. Descriptive statistics were then obtained for all variables and Pearson product moment correlations were calculated between all study variables. When examining the study's first aim, to explore baseline differences between the e-athletes and controls, a multivariate analysis of co-variance (MANCOVA) was used for self-regulation and PYD, and an ANOVA for growth mindset, PA and self-reported health. Because of the potential influence in the developmental process in adolescence, both gender and age were used as co-variates.

To examine the effect of participation in the school esports program, repeated measures ANOVA was conducted. We examined the interaction effect of condition (esports program vs. control) and time (pre- vs. post-test). In addition, we examined the effect of condition (program vs. control) independent of time and time (pre- vs. post-test) independent of condition. Due to COVID-19, the number of individuals completing the post-test data was reduced by 69.15%. As such, age and gender were not included as covariates. In the instance of a significant interaction effect, t-tests were conducted to compare differences. Effect size for the ANOVA was explored using partial eta squared ($\eta_{\text{p}}^{2}$), with a small effect at 0.01–0.059, medium effect 0.06–0.139, and a large effect > 0.14 (Cohen, 1988).

CFA was conducted to explore the psychometric properties of the TSRQ and STQ, but not VSMPYD, using the maximum likelihood method of estimation in AMOS 27 (Arbuckle, 2005). To determine the appropriate fit of the models, the following indices were used: Chi-square statistic (χ^2^), root mean square of approximation (RMSEA: Brown and Cudeck, 1993), Tucker-Lewis index (TLI), Goodness-of Fit Index (GFI), Comparative Fit Index (CFI: Bentler, 1990) and *P* of close fit (Pclose; Hu and Bentler, 1999). The χ^2^ statistics gives an indication of the fit of the data to the model. When the *P* value for χ^2^ is non-significant this indicates a good fit. For the RMSEA, a value of ≤ 0.06 indicates good fit and a value ≤ 0.08 as acceptable (Brown and Cudeck, 1993), when taken together with other indices (Kline, 2011). For the TLI, GFI and CFI, a value ≥0.95 indicates a good fit and ≥0.90 an adequate fit (Hu and Bentler, 1999). Pclose is required to be non-significant (Brown and Cudeck, 1993; Hooper et al., 2008). Finally, reliability analysis was conducted by calculating Cronbach alpha.

## Results

### Baseline Analysis

Means and standard deviations for participant demographics, self-regulation, growth mindset, PYD, PA and sport behaviour, and self-perceived general health are presented in Table 1. 80.1% Of the e-athletes and 70% of the controls indicated that they participated in sport (Chi square = 3.14; *P* = 0.08, Cramer V = 0.13).

**Table 1:** Means and standard deviation for the dependent variables at baseline for the e-athletes and control participants.

	**E-athletes *N* = 89**	**Controls *N* = 99**
TSRQ total	97.2 (13)	96.27 (15.32)
TSRQ meta cognition	47.8 (6.86)	47.27 (7.81)
TSRQ motivation	49.11 (8.67)	49 (8.56)
TSRQ planning	23.47 (4.08)	22.97 (4.32)
TSRQ self-checking	24.32 (3.25)	24.3 (4.8)
TSRQ effort	24.16 (4.54)	24.57 (4.37)
TSRQ self-efficacy	25.26 (4.6)	24.43 (4.79)
Growth mindset	18.05 (3.81)	18.19 (3.54)
PYD competence	8.21 (2.32)	8.89 (1.91)
PYD confidence	8.05 (2.7)	9.14 (2.42)
PYD character	16.05 (1.93)	15.46 (2.26)
PYD caring	12.95 (2.12)	13.38 (1.91)
PYD connection	16.11 (2.4)	15.65 (2.69)
SF1	3.47 (0.96)	3.62 (1.11)
Physical activity	3.94 (1.69)	3.78 (2.01)
Time played Sport (min per week)	253 (159)	351 (227)

The MANCOVA for the four self-regulation factors (Wilk's lambda = 0.98, *P* = 0.57; ηp^2^ = 0.02) and ANCOVA for the higher order factors were all not significant (see Table 2). Similarly, the MANCOVA for PYD (Wilk's lambda = 0.96, *P* = 0.15; ηp^2^ = 0.04) and ANCOVA for growth mindset, PA, and self-perceived general health (see Table 2) were not significant. However, the control group reported significant more minutes of sport participation per week compared to the esports group. Except for sport participation, the results indicate no differences between the students enrolled in the esports program versus the control participants at baseline.

**Table 2:** Mean and standard deviation scores at baseline and post-test.

	**Esports (*****n*** **=** **19)**		**Control (*****n*** **=** **39)**	
	**Pre**	**Post**	**Pre**	**Post**
TSRQ total	97.2 (13)	94.8 (12)	96.3 (15)	97.2 (17)
TSRQ meta cognition	47.8 (6.9)	48.1 (6.2)	47.3 (7.8)	48.3 (8.5)
TSRQ motivation	49.4 (8.7)	46.7 (8.1)	49.0 (8.5)	48.9 (9.2)
TSRQ planning	23.5 (4.1)	24.2 (3.7)	23.0 (4.3)	23.2 (4.5)
TSRQ self-checking	24.3 (3.2)	23.9 (3.2)	24.3 (4.0)	25.1 (4.4)
TSRQ effort	24.2 (4.5)	22.9 (4.1)	24.6 (4.4)	24.4 (4.3)
TSRQ self-efficacy	25.3 (4.6)	23.7 (4.6)	24.4 (4.8)	24.5 (5.1)
Growth mindset	17.4 (3.7)	18.1 (3.8)	17.6 (3.7)	18.2 (3.5)
PYD competence	8.2 (2.3)	8.1 (1.6)	8.9 (1.9)	8.4 (2.2)
PYD confidence	8.1 (2.7)	8.5 (1.8)	9.1 (2.4)	8.6 (2.5)
PYD character	16.0 (1.9)	15.5 (1.8)	15.5 (2.3)	15.6 (2.2)
PYD caring	12.9 (2.1)	12.8 (2.2)	13.4 (1.9)	13.3 (1.9)
PYD connection	16.1 (2.4)	14.8 (3.3)	15.6 (2.7)	15.3 (3.0)
Health (SF1)	3.5 (0.9)	3.1 (0.8)	3.5 (1.1)	3.7 (1.1)
Physical activity	3.2 (1.6)	2.5 (1.8)	3.8 (2.2)	3.5 (2.1)
Time played Sport (min per week)	316 (149)	313 (234)	406 (285)	441 (489)

### Co-variates

There was a significant effect for gender for self-regulation (Wilk's lambda = 0.94, *P* = 0.03; ηp^2^ = 0.06); however, follow-up ANCOVA did not reveal any differences (see Table 2). Gender was also significant for PYD (Wilk's lambda = 0.86, *P* < 0.001; ηp^2^ = 0.15). Sidak *post-hoc* comparisons showed that females scored higher on caring (13.5 vs. 12.5) and the males higher on confidence (9.78 vs. 8.49). The gender effect for PA showed that males were physically active on 4.17 days of the week and females on 3.30, but there were no significant gender differences for growth mindset, sport participation or self-perceived general health.

In terms of age there was a significant effect for self-regulation (Wilk's lambda = 0.89, *P* < 0.001; ηp^2^ = 0.11). *Post-hoc* comparisons showed that the 13–14-year-old group scored higher on all 4 dimensions and higher order factors of self-regulation than the 15–16-year-old group and for the effort factor higher than the 17–18 year old group. The covariate age was also significant for PYD (Wilk's lambda = 0.92, *P* = 0.01; ηp^2^ = 0.09), with the 13–14 year old age group reporting significantly higher levels of competence, confidence and connection than the 15–16 year old group, and also higher competence than the 17–18 year old age group. *Post-hoc* comparisons for growth mindset showed that the youngest group (M = 19.84) scored significantly higher compared to the other two groups (M = 17.62 and M = 15.53, respectively). The 13–14-year old group was also more physically active compared to the other two groups (*P* = 0.04; M = 4.29, 3.58 and 3.06 respectively), but there was no difference for sport participation or self-perceived general health.

### Repeated Measures Analysis

Table 3 provides the means and standard deviations for the dependent variables for those esports (*n* = 19) and control (*n* = 39) students who completed both pre- and post-test instruments, whereas Table 4 provides the results of the repeated measures ANOVAs for the pre-post results of each factor. A significant time main effect was found for PYD connection and PA, indicating a decline for all participants. In addition, there was a significant interaction effect for PYD confidence and a near significant effect for health (medium effect size). For PYD confidence, a paired sample t-test did not show a significant change over time for the esports conditions (*t* = −0.89; *P* = 0.39), but the control group showed a significant decline (*t* = 2.42; *P* = 0.02). For health, the change from baseline to post-test was not significant for either group (*P* > 0.10).

**Table 3:** Results of the repeated measures analysis of variance.

	**Time *F*(1, 54)**	**Condition *F*(1, 54)**	**Time × condition F (1, 54)**
TSRQ total	0.23; *P* = 0.64; $\eta_{\text{p}}^{2}$ < 0.01	0.04; *P* = 0.85; $\eta_{\text{p}}^{2}$ < 0.01	1.23; *P* = 0.27; $\eta_{\text{p}}^{2}$ = 0.02
TSRQ meta cognition	0.51; *P* = 0.48; $\eta_{\text{p}}^{2}$ = 0.01	0.01; *P* = 0.94; $\eta_{\text{p}}^{2}$ < 0.01	0.15; *P* = 0.70; $\eta_{\text{p}}^{2}$ < 0.01
TSRQ motivation	3.26; *P* = 0.08; $\eta_{\text{p}}^{2}$ = 0.06	0.15; *P* = 0.70; $\eta_{\text{p}}^{2}$ < 0.01	2.81; *P* = 0.10; $\eta_{\text{p}}^{2}$ = 0.05
TSRQ planning	0.59; *P* = 0.45; $\eta_{\text{p}}^{2}$ = 0.01	0.48; *P* = 0.49; $\eta_{\text{p}}^{2}$ = 0.01	0.13; *P* = 0.72; $\eta_{\text{p}}^{2}$ < 0.01
TSRQ Self-checking	0.21; *P* = 0.65; $\eta_{\text{p}}^{2}$ < 0.01	0.33; *P* = 0.57; $\eta_{\text{p}}^{2}$ = 0.01	1.46; *P* = 0.23; $\eta_{\text{p}}^{2}$ = 0.03
TSRQ effort	2.35; *P* = 0.13; $\eta_{\text{p}}^{2}$ = 0.04	0.67; *P* = 0.41; $\eta_{\text{p}}^{2}$ = 0.01	1.50; *P* = 0.23; $\eta_{\text{p}}^{2}$ = 0.03
TSRQ Self-efficacy	2.41; *P* = 0.13; $\eta_{\text{p}}^{2}$ = 0.04	0.01; *P* = 0.98; $\eta_{\text{p}}^{2}$ < 0.01	2.77; *P* = 0.10; $\eta_{\text{p}}^{2}$ = 0.05
Growth MINDSET	1.38; *P* = 0.25; $\eta_{\text{p}}^{2}$ = 0.03	0.04; *P* = 0.85; $\eta_{\text{p}}^{2}$ < 0.01	0.01; *P* = 0.96; $\eta_{\text{p}}^{2}$ < 0.01
PYD competence	1.72; *P* = 0.20; $\eta_{\text{p}}^{2}$ = 0.03	0.98; *P* = 33; $\eta_{\text{p}}^{2}$ = 0.02	0.45; *P* = 0.51; $\eta_{\text{p}}^{2}$ = 0.01
PYD confidence	0.10; *P* = 0.76; $\eta_{\text{p}}^{2}$ < 0.01	0.84; *P* = 0.36; $\eta_{\text{p}}^{2}$ = 0.02	4.43; *P* = 0.04; $\eta_{\text{p}}^{2}$ = 0.08
PYD character	0.50; *P* = 0.48; $\eta_{\text{p}}^{2}$ = 0.01	0.19; *P* = 0.67; $\eta_{\text{p}}^{2}$ < 0.01	1.59; *P* = 0.31; $\eta_{\text{p}}^{2}$ = 0.03
PYD caring	0.12; *P* = 0.73; $\eta_{\text{p}}^{2}$ < 0.01	0.83; *P* = 0.37; $\eta_{\text{p}}^{2}$ = 0.02	0.01; *P* = 0.99; $\eta_{\text{p}}^{2}$ < 0.01
PYD connection	7.90; *P* = 0.01; $\eta_{\text{p}}^{2}$ = 0.13	0.01; *P* = 0.97; $\eta_{\text{p}}^{2}$ < 0.01	2.64; *P* = 0.11; $\eta_{\text{p}}^{2}$ = 0.05
Health: SF1	0.62; *P* = 0.44; $\eta_{\text{p}}^{2}$ = 0.01	1.55; *P* = 0.22; $\eta_{\text{p}}^{2}$ = 0.03	3.65; *P* = 0.06; $\eta_{\text{p}}^{2}$ = 0.06
Physical activity	3.86; *P* = 0.05; $\eta_{\text{p}}^{2}$ = 0.06	2.59; *P* = 0.11; $\eta_{\text{p}}^{2}$ = 0.04	0.56; *P* = 0.46; $\eta_{\text{p}}^{2}$ = 0.01
Sport played	0.08; *P* = 0.78; $\eta_{\text{p}}^{2}$ < 0.01	1.20; *P* = 0.28; $\eta_{\text{p}}^{2}$ = 0.03	0.11; *P* = 0.74; $\eta_{\text{p}}^{2}$ < 0.01

*Time = pre vs. post; Condition = Control vs. Esports. The grey shaded areas highlight mean P < 0.05 or a medium effect size*.

**Table 4:** Results of the multivariate analysis of covariance (grey area indicate significant effect).

	**ANCOVA: F value, *P* value and Effect Size**	**Covariate Gender: F value, *P* value and Effect Size**	**Covariate Age: F value, *P* value and Effect Size**
TSRQ total	2.35; *P* = 0.13; $\eta_{\text{p}}^{2}$ = 0.01	0.05; *P* = 0.82; $\eta_{\text{p}}^{2}$ < 0.001	14.8; *P* < 0.001; $\eta_{\text{p}}^{2}$ = 0.08
TSRQ meta cognition	2.29; *P* = 0.13; $\eta_{\text{p}}^{2}$ = 0.01	0.01; *P* = 0.98; $\eta_{\text{p}}^{2}$ < 0.001	8.61; *P* = 0.004; $\eta_{\text{p}}^{2}$ = 0.05
TSRQ motivation	1.80; *P* = 0.18; $\eta_{\text{p}}^{2}$ = 0.01	0.14; *P* = 0.71; $\eta_{\text{p}}^{2}$ = 0.01	17.4; *P* < 0.001; $\eta_{\text{p}}^{2}$ = 0.09
TSRQ planning	1.77; *P* = 0.19; $\eta_{\text{p}}^{2}$ = 0.01	0.02; *P* = 0.90; $\eta_{\text{p}}^{2}$ < 0.001	8.32; *P* = 0.004; $\eta_{\text{p}}^{2}$ = 0.04
TSRQ self-checking	2.37; *P* = 0.13; $\eta_{\text{p}}^{2}$ = 0.01	0.03; *P* = 0.85; $\eta_{\text{p}}^{2}$ < 0.001	7.08; *P* = 0.01; $\eta_{\text{p}}^{2}$ = 0.04
TSRQ effort	1.13; *P* = 0.29; $\eta_{\text{p}}^{2}$ = 0.01	2.33; *P* = 0.13; $\eta_{\text{p}}^{2}$ = 0.01	21.6; *P* < 0.001; $\eta_{\text{p}}^{2}$ = 0.11
TSRQ self-efficacy	1.99; *P* = 0.16; $\eta_{\text{p}}^{2}$ = 0.01	0.44; *P* = 0.51; $\eta_{\text{p}}^{2}$ = 0.002	10.4; *P* = 0.001; $\eta_{\text{p}}^{2}$ = 0.05
Growth mindset	0.01; *P* = 0.98; $\eta_{\text{p}}^{2}$ < 0.001	0.79; *P* = 0.38; $\eta_{\text{p}}^{2}$ = 0.004	22.4; *P* < 0.001; $\eta_{\text{p}}^{2}$ = 0.11
PYD competence	0.13; *P* = 0.72; $\eta_{\text{p}}^{2}$ = 0.001	1.70; *P* = 0.19; $\eta_{\text{p}}^{2}$ = 0.01	12.1; *P* < 0.001; $\eta_{\text{p}}^{2}$ = 0.06
PYD confidence	2.01; *P* = 0.16; $\eta_{\text{p}}^{2}$ = 0.01	17.1; *P* < 0.001; $\eta_{\text{p}}^{2}$ = 0.09	6.41; *P* = 0.01; $\eta_{\text{p}}^{2}$ = 0.03
PYD character	1.49; *P* = 0.22; $\eta_{\text{p}}^{2}$ = 0.01	4.03; *P* = 0.05; $\eta_{\text{p}}^{2}$ = 0.02	1.83; *P* = 0.18; $\eta_{\text{p}}^{2}$ = 0.01
PYD caring	0.43; *P* = 0.51; $\eta_{\text{p}}^{2}$ = 0.002	4.46; *P* = 0.04; $\eta_{\text{p}}^{2}$ = 0.02	5.31; *P* = 0.02; $\eta_{\text{p}}^{2}$ = 0.03
PYD connection	0.41; *P* = 0.53; $\eta_{\text{p}}^{2}$ = 0.002	0.38; *P* = 0.54; $\eta_{\text{p}}^{2}$ = 0.002	7.05; *P* = 0.01; $\eta_{\text{p}}^{2}$ = 0.04
Health: SF1	3.22; *P* = 0.07; $\eta_{\text{p}}^{2}$ = 0.02	0.63; *P* = 0.43; $\eta_{\text{p}}^{2}$ = 0.003	0.01; *P* = 0.95; $\eta_{\text{p}}^{2}$ < 0.001
Physical activity	0.30; *P* = 0.59; $\eta_{\text{p}}^{2}$ = 0.002	8.93; *P* = 0.003; $\eta_{\text{p}}^{2}$ = 0.05	10.0; *P* = 0.002; $\eta_{\text{p}}^{2}$ = 0.05
Sport played	8.73; *P* = 0.004; $\eta_{\text{p}}^{2}$ = 0.06	2.52; *P* = 0.12; $\eta_{\text{p}}^{2}$ = 0.02	2.87; *P* = 0.09; $\eta_{\text{p}}^{2}$ = 0.02

## Discussion

This study aimed to (i) compare adolescent esports athletes with aged-matched controls on self-regulation, growth mindset, PYD, PA and sport participation, and self-perceived general health, and (ii) examine how these factors changed following participation in a school esports programme. The findings suggested that there were no differences between the e-athletes and controls on any of the variables at baseline, except for sport participation (higher in control participants). Both the e-athletes and control participants showed a decrease in PYD connection and PA behaviour over the program period. In addition, the control group showed a decline in PYD confidence, whereas the esports group did not.

### Self-Regulation

Results indicated no significant differences between control participants and e-athletes at baseline or after involvement in the school esports program. Previous research, albeit in adults and using a different instrument, has shown that e-athletes had significantly lower levels of self-regulation compared to traditional sport athletes (Trotter et al., 2021). It is possible that the e-athletes developed similar self-regulatory skills compared to the control individuals in the school or sport settings. For example, prior to enrolment to the esports school program, 81.1% of the e-athletes were engaged in sport compared to 70% of the controls. As such, the co-regulation of psychological skills (e.g., goal setting, self-reflection) associated with self-regulatory behaviours might have been developed in the e-athletes prior to the esports program enrolment.

During the COVID-19 lockdown schools closed between the 26th of March, 2020 and the 25th May 2020 (Australian Broadcasting Commission, 2020). During the lockdown, interactions with coaches, teachers, or peers, often required for the development of self-regulatory skills (Collins and Durand-Bush, 2014), were not possible face-to-face, but only online. It is unclear whether online interactions have similar impact as those which happen face-to-face. Both the frequency and quality of interactions was likely influenced by the COVID19 lockdown during this period. Future research would need to explore the efficacy of online interactions on the development of self-regulatory skills.

### Growth Mindset

No statistically significant difference between the e-athletes and control participants on growth mindset was found. Previous research from traditional sports literature has suggested that a growth mindset is important for elite athlete development alongside optimism and proactively seeking feedback from coaches (Lauer et al., 2018). Interventions have been shown to be an effective method for increasing the growth mindset of high school students (Yeager et al., 2020). However, to date, there has not been research determining if participation in traditional sports developmental programs innately develops a growth mindset. Similarly, there is no research in esports performance literature that has explored the role of esports developmental programs effectiveness of increasing participants' growth mindset. It is likely that more specific interventions are required to improve an e-athletes growth mindset to enhance self-confidence and development of in-game knowledge, as suggested in previous literature (Himmelstein et al., 2017).

### Positive Youth Development

At baseline, no differences were found for PYD between the e-athletes and control participants. However, there was a time main effect for connection and interaction effect for confidence. Although playing video games has been shown to enhance a sense of belonging and increase relationships with family and friends (Vella et al., 2017), not surprisingly connection decreased in both groups likely because of the COVID-19 lockdown in Queensland during the study period. This allowed only for virtual meetings and prohibited face-to-face meetings with peers, teachers, family, or friends. Several studies have found that lockdowns resulted in increased isolation and loneliness (e.g., Burrai et al., 2021). Loneliness during the COVID-19 lockdown has been associated with several negative mental health outcomes, such as depression and suicide, and has remained elevated despite the easing of lockdowns (Killgore et al., 2020). Despite this, it has been shown that social connection through technology (e.g., social media) is a protective factor of loneliness during lockdowns (Cauberghe et al., 2021; Okabe-Miyamoto and Lyubomirsky, 2021). The results from this study contradict previous studies that suggest technology is a protective factor to the negative psychological outcomes of lockdown (Cauberghe et al., 2021). This finding suggests that face-to-face esports training is possibly more important to increase connectedness than online training.

In terms of confidence, the control group showed a decline whereas the e-athletes remained at a similar level (despite a slight increase in confidence scores from 8.1 to 8.5). An explanation for the decline in the control group is the notion that their confidence levels were higher (albeit not significantly) at baseline. Ultimately, the esports and control groups had similar values for confidence at the post-test timepoint. An alternative explanation is that the COVID19 lockdown resulted in the reduction in confidence for both groups. This might have been due to a reduction in social interactions and engaging mainly in novel online learning environments.

### Perceived General Health

The *post-hoc* comparisons for health did not reveal differences between the two groups. Overall, the score for perceived general health was only between fair and good at both baseline and post-test. At baseline, 43% of participants reported health to be poor, fair, or good. Previous research has suggested that an individual's perception of illness is directly associated with the likelihood of behaviour change (Champion and Skinner, 2008). A recent study has indicated that a lack of understanding how adolescents perceive their health is likely to lead to unsuccessful interventions (Ott et al., 2011). Previous research has indicated various health issues related to esports participation (Zwibel et al., 2019). Yet, the lack of a significant difference between the e-athletes and control participants may indicate that youth e-athletes do not perceive any negative health outcomes to be associated with esports participation, or that participation in esports do not have a significant impact on their health.

### Physical Activity and Sport Participation

There was no baseline or post-test difference in PA behaviour between the e-athletes and controls. However, there was a significant decrease for both groups from baseline to post-test. This decrease in PA behaviour was likely due to the COVID-19 restrictions. Research investigating PA levels in Irish adolescents (*n* = 1214) during the COVID-19 lockdown showed that half of the adolescents reported being less physically active, compared to a third who did the same, and only a fifth who were more physically active (Ng et al., 2020). These findings were replicated by Stockwell et al. (2021) in a systematic review of PA and sedentary behaviour before and during the COVID-19 lockdown. They found that more than 50% of the population of both healthy adults and children's PA levels decreased, despite government organisations providing guidance on staying physically active during lockdown.

Although the percentage of e-athletes who participated in sport was higher than controls, the control group reported more minutes per week in sport. One possible explanation is that the e-athletes engaged in playing esports prior to enrolment in the school esports program, leaving less leisure time for engagement in organised sport. In future research, more detailed information is required on how adolescents use their leisure time. In addition, it was noticeable that there was significant variability in the minutes participated in sport each week in both the e-athletes and controls.

Previously, it has been suggested that a benefit of co-curricular esports programs could increase the PA levels, including sport participation, of students (Shum et al., 2021). This suggestion is supported by recent research which found that high school esports programs in Europe have been reported to include PA as part of regular training (Tjønndal and Skauge, 2020; Wimmer et al., 2021). Norwegian high schools have even included PA and physical conditioning as part of the learning objectives of their program, arguing that an understanding of nutrition, rest and recovery are important to youth e-athlete development. Similarly, teachers involved in the esports program, in the present study, included PA as part of regular training and as a reward for participation in esports tournaments. Of course, this was not implemented because of the COVID-19 restrictions. However, the promotion of PA or sport participation as part of adolescent developmental esports programs (Tjønndal and Skauge, 2020; Shum et al., 2021) might help to enhance physical fitness in e-athletes.

### Role of Gender and Age

Despite no baseline differences being found, significant covariate effects were found for gender and age. In line with previous research, the males were more active (Gortmaker et al., 2012), scored higher on PYD confidence (O'Connor et al., 2020), but lower on PYD caring (Conway et al., 2015) compared to the females. The gender differences in PYD have been attributed to females providing increased social contributions (e.g., more support to friends and family) and higher school engagement (more perceived performance, less boredom) (Gomez-Baya et al., 2021).

The covariate age was significantly related to self-regulation, positive youth development, growth mindset and PA, but not sport participation or self-reported general health. The finding that self-regulations was highest in the younger group contradicts previous findings that self-regulation increases with age at the behavioural and neurological levels (Steinberg et al., 2008; Monahan et al., 2009; King et al., 2013). A possible explanation for this finding might be the suitability of the TSRQ for younger adolescents. Similarly, the young group scored higher on PYD confidence and connection compared to the 15–16-year-old group, and higher on competence compared to both older groups. In terms of connectedness, this finding is not surprising, since research has shown that social connectedness decreases with age (Cole and Kerns, 2001). In addition, developmental experiences and plasticity in development might result in adolescents being pulled in different trajectories and having different developmental pathways (Larson and Tran, 2014).

The youngest group also had the highest score for growth mindset. This finding is in line with results suggesting that adolescents' growth mindset decreases as they age. Also, cognitive interventions to improve growth mindset are most effective when targeting young people and old people, but less effective with adolescents (Guye et al., 2017). Finally, as expected, PA levels decreased with age (Gordon-Larsen et al., 2004; Gortmaker et al., 2012).

### Study Limitations and Future Research

This study has several limitations. The first limitation of this study was the unexpected COVID-19 virus and the lockdown and quarantine which resulted in significant participant drop-out. Due to the substantial dropout of participants, it not possible to determine the full extent of the COVID-19 lockdown on participants health, psychology and wellbeing. Future research should explore the impact of health, psychology, and wellbeing of adolescent e-athletes uninterrupted by a global pandemic. Secondly, this study was conducted using only self-report measures. Participants may have responded to questions in a way in which they believe is socially desirable. Furthermore, future research could employ more objective methods of measuring PA and sport behaviour using pedometers or accelerometers, as well as physical fitness measures (e.g., VO_2_max). Additionally, the measures used in this study had different numbers of anchors in their Likert scales. Constantly switching between the numbers of anchors a scale might impact the cognitive effort required to complete surveys. Future studies might consider standardising Likert scales to reduce participant cognitive effort. Another limitation is the current state of organisation of high school esports programs in Australia. High school esports programs in Australia are still in their infancy and unregulated. The potential differences in the esports programs across the 7 schools also represents a limitation to this study. As different schools are at different stages of development, it is possible that students had different experiences. Future research should continue to evaluate the practises of high school esports programs. The consistency of the findings will improve as youth esports programs become more established.

## Conclusion

This study investigated the influence of participation in an esports school program on the psychological (self-regulation, growth-mindset, and positive youth development) and health (PA, sport participation, self-perceived general health) factors of adolescent e-athletes and aged-matched control participants. A novel finding was that, at baseline, there were no differences between those enrolled in the esports program and controls, except for sport participation. Both conditions reported decreased PYD and PA behaviours. However, the control group showed a decrease in PYD confidence, while the esports group did not. These changes were most likely due to COVID19 lockdowns in Queensland during the study period. Overall, school esports programs appear to attract adolescents who are similar in their psychological functioning and health compared to their peers. In addition, the school esports programs have the potential to bring about positive psychological development and/or health behaviour change, if implemented appropriately (Polman et al., 2018).

## Data Availability Statement

The raw data supporting the conclusions of this article will be made available by the authors, without undue reservation.

## Ethics Statement

The studies involving human participants were reviewed and approved by QUT Human Ethics Advisory Team. Written informed consent from the participants' legal guardian/next of kin was not required to participate in this study in accordance with the national legislation and the institutional requirements.

## Author Contributions

MT, RP and TC: methodology, investigation, and writing—original draft preparation. MT, RP, and DP: formal analysis and resources. RP: data curation. MT: writing—review and editing. TC, PD, and RP: supervision. TC, PD, and RP: project administration. All authors have read and agreed to the published version of the manuscript.

## Conflict of Interest

The authors declare that the research was conducted in the absence of any commercial or financial relationships that could be construed as a potential conflict of interest.

## Publisher's Note

All claims expressed in this article are solely those of the authors and do not necessarily represent those of their affiliated organizations, or those of the publisher, the editors and the reviewers. Any product that may be evaluated in this article, or claim that may be made by its manufacturer, is not guaranteed or endorsed by the publisher.
